# PRBS Gas Challenges Reveal Impaired Chemoreflex and Cholinergic Dynamics in MCI

**DOI:** 10.1007/s10439-026-04213-7

**Published:** 2026-06-10

**Authors:** Suhaib Hashem, Stanley Yamashiro, Elizabeth Joe, Helena Chui, Vasilis Marmarelis

**Affiliations:** 1 Alfred E. Mann Department of Biomedical Engineering, University of Southern California, Los Angeles, CA USA; 2 Department of Neurology, Keck School of Medicine of USC, Los Angeles, CA USA

**Keywords:** Cerebral autoregulation, Pseudorandom binary sequence (PRBS), Principal dynamic mode (PDM), Mild cognitive impairment (MCI), Chemoreflex sensitivity, Alzheimer’s disease

## Abstract

**Purpose:**

This study investigates the dynamic regulation of cerebral oxygenation in individuals with mild cognitive impairment (MCI) compared to healthy controls, using a novel pseudorandom binary sequence (PRBS) gas challenge. Traditional assessments often overlook frequency-dependent features of cerebrovascular control; this work aims to uncover latent deficits through broadband perturbations and nonparametric dynamic modeling.

**Methods:**

Seventeen ApoE4-negative participants (9 controls, 8 MCI) underwent a three-session supine protocol involving PRBS-modulated inhalation of hypoxic and hypercapnic gas mixtures. Physiological signals—including near-infrared spectroscopy (TOI), arterial pressure (ABP), and end-tidal CO_2_ (PETCO_2_)—were recorded. Laguerre-based Volterra modeling and Principal Dynamic Mode (PDM) decomposition were used to analyze the dynamic response of cerebral oxygenation to ABP and CO_2_ stimuli. Group differences were statistically assessed using Welch’s *t*-tests and repeated measures ANOVA.

**Results:**

Controls showed significant increases in ventilation and TOI from baseline to stimulation ($p < 0.01$), while MCI did not. Gain of the first CO_2_ PDM increased in controls during PRBS (*p* = 0.016) but decreased in MCI. A low-frequency ABP-derived PDM ($\sim$ 0.014 Hz), consistent with endothelial-dependent vasodilation, was elevated only in controls during stimulation. Persistent differences in spectral recovery, kernel profiles, and PDM Gains suggested impaired baroreflex and chemoreflex regulation, and possible disruption of cholinergic-linked neurovascular coupling in MCI.

**Conclusion:**

PRBS gas modulation combined with dynamic modeling revealed subtle but significant cerebrovascular control impairments in MCI. This methodology enables mechanistic insights into early pathophysiology and may aid future physiomarker development.

## Introduction

Cerebral oxygenation dynamics are regulated by the integrated effects of vascular reactivity, metabolic demand, and systemic blood gas levels [5, 7, 11, 13]. In dementia and prodromal stages such as Mild Cognitive Impairment (MCI), disruptions in these regulatory mechanisms have been widely reported [12, 20, 24, 30]. Specifically, Heutz et al. [12] reported inconsistent findings in dynamic cerebral autoregulation across methods and dementia subtypes, highlighting the need for standardized perturbation-based approaches. McKetton et al. [20] demonstrated elevated cerebrovascular resistance in MCI using BOLD-CVR mapping, while Tomoto et al. [28] found impaired CO$_2$ vasomotor reactivity in amnestic MCI using transcranial Doppler. Most studies to date have assessed these impairments under spontaneous or steady-state conditions, limiting the ability to resolve frequency-dependent features of the response [17, 20, 24]. Although deficits in cerebral autoregulation and vasomotor reactivity have been identified, their dynamic characterization under controlled perturbations, such as hypoxic and hypercapnic breathing, remains largely unexplored.

Pseudorandom binary sequence (PRBS) stimuli were first introduced into physiological system identification by Marmarelis to enable broadband probing of system dynamics across a wide frequency range [21]. PRBS inputs possess a flat spectral profile, allowing for uniform excitation of system dynamics and improving the robustness of model estimation compared to relying solely on spontaneous fluctuations. Subsequent applications demonstrated the utility of PRBS in ventilatory control experiments, enabling the separation of overlapping regulatory mechanisms such as chemoreflex and baroreflex contributions [4, 8, 10]. Unlike traditional step, ramp, or cyclic stimuli, PRBS allows simultaneous excitation of both fast and slow dynamic components within a single experimental session. A comprehensive treatment of the mathematical foundations underlying these techniques, including the Laguerre Expansion Technique and Volterra kernel estimation, is provided in Marmarelis [19].

Hypoxic and hypercapnic challenges modulate distinct physiological pathways involved in cerebral blood flow regulation. Hypoxia primarily stimulates peripheral chemoreceptors located in the carotid bodies, while hypercapnia activates both peripheral and central chemoreceptors, influencing cerebral vascular tone [14, 25]. In addition to chemoreceptive effects, both stimuli have been shown to impact baroreflex sensitivity and autonomic control, further complicating the integrated regulation of cerebral oxygenation [1, 31]. In this study, a PRBS breathing protocol was used to modulate these inputs dynamically, providing a unique opportunity to assess their combined and independent effects on cerebral oxygenation in both healthy and cognitively impaired populations.

Cerebrovascular regulatory mechanisms operate across distinct frequency bands: endothelial and nitric oxide-dependent processes reside below 0.02 Hz, neurogenic and cholinergic regulation at approximately 0.02–0.04 Hz, myogenic autoregulation at 0.04–0.15 Hz, and respiratory-cardiac influences above 0.15 Hz [7, 16]. A perturbation method that excites all of these bands simultaneously is therefore essential for characterizing the full spectrum of cerebrovascular control.

Building on our prior use of the Laguerre Expansion Technique (LET) to model cerebral oxygenation and identify impairments in Alzheimer’s disease [22, 24], this study applies Principal Dynamic Mode (PDM) analysis to compare oxygenation dynamics in MCI and control groups. PDM analysis builds on kernel-based modeling by extracting distinct dynamic patterns with characteristic spectral profiles, offering a clearer view of the underlying physiological mechanisms. Unlike subject-specific kernel estimates, PDMs provide a standardized representation that enables meaningful comparisons across individuals and experimental groups.

This study represents the first application of PRBS-induced hypoxic and hypercapnic breathing, combined with nonparametric dynamic modeling, to assess group differences in cerebrovascular regulation. By introducing broadband perturbations and using PDM analysis, we aim to establish a methodological framework capable of understanding and detecting early impairments in cerebral oxygenation control that may precede the onset of dementia. Through this approach, we aim to identify early mechanistic deficits that may underlie the progression from MCI to dementia.

## Materials and Methods

### Subject Demographics

A total of 17 participants (9 controls, 8 MCI) who do not carry the ApoE4 gene were recruited for this study. All participants provided written informed consent prior to enrollment. The study protocol was approved by the Institutional Review Board (IRB) at the University of Southern California. Diagnosis was based on the Clinical Dementia Rating (CDR) obtained prior to data collection. Subjects were screened to exclude major cardiorespiratory, cerebrovascular, or unrelated neurological conditions. Participants were recruited from a larger NIA-funded longitudinal cohort (R01-AG058162) at the University of Southern California. The requirement for ApoE4-negative genotype further constrained the eligible pool, as ApoE4 carriers were excluded to isolate effects of cognitive status from the known cerebrovascular impact of the ApoE4 allele [18]. Although the resulting sample size is modest, it is consistent with pilot investigations of novel perturbation-based paradigms in this population and is sufficient for the exploratory, hypothesis-generating aims of this study.

### PRBS Gas Delivery Setup

**Fig. 1:**
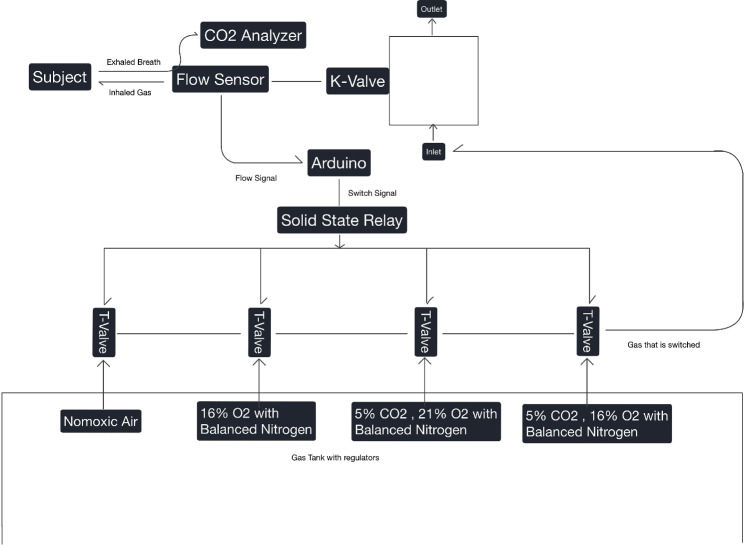
Schematic of PRBS setup

To assess cerebral oxygenation dynamics, we developed a custom gas delivery device employing pseudorandom binary sequence (PRBS)-modulated hypoxic and hypercapnic stimuli [4]. Subjects breathed through a disposable thermoplastic mouthpiece connected to a pneumotachograph measuring expiratory airflow. Real-time detection of exhalation onset, via an Arduino Uno microcontroller, triggered gas switching to synchronize precisely with the respiratory cycle (Fig. 1).

Two independent PRBS signals, each initialized with distinct seeds, controlled four solenoid valves through a four-channel solid-state relay. Each valve connected via T-junctions to a distinct gas mixture. An 8-seed PRBS sequence yielded $2^8 - 1 = 255$ transitions over a 15-min session which approximately matches a breathing frequency of 14–18 breaths per minute.

### Physiological Measurements

Physiological signals were recorded continuously at 500 Hz using:

- **Near-Infrared Spectroscopy (Hamamatsu NIRO-300)** for cortical tissue oxygenation (TOI).
- **Non-invasive Blood Pressure Monitor (NIBP100D)** for arterial blood pressure (ABP).
- **Capnograph (CapnoTek)** for end-tidal CO_2_ (PETCO_2_).
- **Pulse Oximeter (Nonin 7000 series)** for heart rate and SpO_2_.
- **Pneumotachograph (SpiroQuant H)** to capture airflow and trigger PRBS switching.

Beat-to-beat signals (HR, ABP, SpO_2_) and breath-to-breath signals (PETCO_2_, tidal volume) were extracted. Tidal volume was calculated as the integral of exhaled flow per breath. Ventilation was computed breath-by-breath as:

$$V_E = \frac{\text {tidal volume}}{\text {inhalation time} + \text {exhalation time}}$$

All signals were visually inspected for motion artifacts and sensor dropouts; no subjects were excluded due to data quality. Signals were then mean-subtracted and high-pass filtered at 0.005 Hz to remove slow non-stationary drift, and resampled to a common 2 Hz grid via cubic spline interpolation to align all channels temporally. The 2 Hz resampling rate provides greater than 4$\times$ Nyquist margin for the cerebrovascular dynamics of interest, which reside predominantly below 0.5 Hz [7]. ABP and NIRS signals were temporally aligned through the common acquisition system and resampling grid; no additional physiological landmark-based alignment was applied.

### Experimental Protocol

Participants completed three consecutive 10–15 min sessions in the supine position:

- **Session 1 (Baseline)** Ambient air breathing (10 min)
- **Session 2 (PRBS)** Stimulated gas switching using PRBS (15 min)
- **Session 3 (Post)** Recovery with ambient air (10 min)

Gas composition per PRBS state:

- Mixture 1: 21% O_2_, 0% CO_2_ (ambient)
- Mixture 2: 16% O_2_, 0% CO_2_
- Mixture 3: 21% O_2_, 5% CO_2_
- Mixture 4: 16% O_2_, 5% CO_2_

### Modeling Methodology

We used the Volterra series framework with the Laguerre Expansion Technique (LET) to model the relationship between inputs (ABP, PETCO_2_) and output (TOI). The first-order model is expressed as:

1 $$\begin{aligned} y(t) = k_0 + \sum _{\tau =0}^{M} k_{\text {ABP}}(\tau ) \, p(t - \tau ) + \sum _{\tau =0}^{M} k_{\text {CO}_2}(\tau ) \, x(t - \tau ) + \epsilon (t) \end{aligned}$$

where *y*(*t*) is TOI, *p*(*t*) is ABP, *x*(*t*) is PETCO_2_, $k_0$ is the baseline offset, and $\epsilon (t)$ is the residual [19]. Each input was convolved with Laguerre basis functions, selecting 5 for ABP and 4 for CO_2_ based on Bayesian Information Criterion (BIC) minimization. The Laguerre rate parameter $\alpha$ was set to 0.55 for ABP and 0.85 for CO$_2$. The memory length *M* for each input was computed as $M = \text {round}(-30/\ln \alpha )$, which ensures sufficient time for the Laguerre basis functions to relax, yielding $M = 50$ samples (25 s) for ABP and $M = 185$ samples (92.5 s) for CO$_2$ at the 2 Hz sampling rate. The longer CO$_2$ memory accommodates the slower time constants of chemoreflex-mediated cerebrovascular dynamics [19]. Results were verified to be stable across neighboring model orders (4–6 for ABP, 3–5 for CO$_2$).

#### Estimation via Ordinary Least Squares

After Laguerre transformation, the model reduces to:

2 $$\begin{aligned} \textbf{y} = \textbf{V} \textbf{c} + \boldsymbol{\epsilon } \end{aligned}$$

where $\textbf{V}$ is the Laguerre-transformed input matrix, $\textbf{y}$ is the TOI vector, and $\textbf{c}$ are the coefficients estimated via:

3 $$\begin{aligned} \textbf{c} = (\textbf{V}^T \textbf{V})^{-1} \textbf{V}^T \textbf{y} \end{aligned}$$

#### Model Validation

Model prediction accuracy was assessed using normalized mean square error (NMSE):

4 $$\begin{aligned} \text {NMSE} = 1 - \frac{\sum _t \left( y(t) - \hat{y}(t) \right) ^2}{\sum _t \left( y(t) - \bar{y} \right) ^2} \end{aligned}$$

### Principal Dynamic Mode (PDM) Analysis

To reduce model complexity and enhance interpretability, we decomposed the first-order kernels into orthonormal dynamic modes via singular value decomposition (SVD) [19]. For each input (ABP and PETCO_2_) independently, a kernel matrix $\textbf{K}$ was formed by row-concatenating all control-group baseline session kernels. SVD was applied: $\textbf{K} = \textbf{U} \boldsymbol{\Sigma } \textbf{V}^T$, where the columns of $\textbf{V}$ are the PDMs. The number of retained PDMs was determined by the number of Laguerre functions used in the initial kernel modeling. PDMs were extracted from all baseline session kernels in the control group and used to project all subject-specific kernels. Subject-specific Gains were computed by projecting each kernel onto the PDM basis: $G_j = \textbf{k}_i^T \cdot \boldsymbol{\phi }_j$. Each kernel was then expressed as:

5 $$\begin{aligned} k_i(\tau ) = \sum _{j=1}^{J} G_j \cdot \phi _j(\tau ) \end{aligned}$$

where $\phi _j(\tau )$ is the *j*-th PDM and $G_j$ is the associated Gain. These Gains quantify the contribution of each physiological mechanism and serve as group-comparable features.

### Statistical Analysis

All physiological metrics and model-derived parameters were compared between groups using Welch’s *t*-tests for session-specific comparisons. To assess within-subject changes across sessions and group-by-session interactions, we performed repeated-measures ANOVA (RM-ANOVA) with Group (Control vs. MCI) as a between-subjects factor and Session (1, 2, 3) as a within-subjects factor. Post hoc pairwise comparisons used paired *t*-tests within each group. To address the possibility that baseline PETCO_2_ differences between groups could confound PDM Gain comparisons, we note that: (a) PETCO_2_ enters the model as an explicit input and signals are mean-subtracted prior to estimation, removing baseline operating-point effects by design; (b) RM-ANOVA on raw PETCO_2_ showed a non-significant group main effect ($p = 0.071$) and non-significant interaction ($p = 0.554$). Statistical significance was set at $p < 0.05$.

## Results

The means and standard deviations of heart rate, oxygen saturation, ventilation, mean arterial blood pressure (ABP), end-tidal CO_2_ (PETCO_2_), tissue oxygenation index (TOI), and model NMSE values for each session are presented in Table 1. Across all sessions, none of the group-wise mean values were significantly different. Repeated-measures ANOVA revealed a significant main effect of Session for PETCO_2_ ($p = 2.47 \times 10^{-8}$) and ventilation ($p = 0.003$), with no significant Group $\times$ Session interactions for PETCO_2_ ($p = 0.554$) or ventilation ($p = 0.081$). A significant session effect for SpO_2_ ($p = 0.036$) was driven by lower baseline SpO_2_ in MCI subjects.

**Table 1:** Means and standard deviations of heart rate (HR), oxygen saturation (SpO_2_), ventilation (Ve), mean arterial blood pressure (ABP), end-tidal CO_2_ (PETCO_2_), tissue oxygenation (TOI), and normalized mean square error (NMSE) for controls and MCI

		Controls		MCI		*p*-value
		Mean	SD	Mean	SD	
Heart rate	Session 1	61.8	10.37	68.22	9.73	0.208
	Session 2	62.16	9.82	66.7	8.58	0.325
	Session 3	60.45	10.39	66.45	8.84	0.218
SpO_2_	Session 1	94.87	1.58	93.12	2.39	0.105
	Session 2	95.11	1.63	94.81	1.00	0.655
	Session 3	95.11	2.12	94.82	1.34	0.744
Ventilation	Session 1	8.22	1.02	9.25	1.81	0.180
	Session 2	9.30	1.13	9.43	1.54	0.845
	Session 3	8.61	1.12	8.84	1.49	0.733
ABP	Session 1	102.86	20.15	105.86	14.87	0.730
	Session 2	110.37	15.01	106.56	10.21	0.546
	Session 3	107.48	15.89	111.11	19.28	0.680
PETCO_2_	Session 1	35.20	1.97	32.35	4.36	0.121
	Session 2	40.78	3.04	38.27	3.14	0.116
	Session 3	35.24	2.62	33.65	4.42	0.394
TOI	Session 1	69.83	5.06	67.01	7.30	0.207
	Session 2	71.02	4.71	68.79	4.52	0.169
	Session 3	69.87	5.34	66.96	5.15	0.117
NMSE	Session 1	0.68	0.13	0.67	0.17	0.921
	Session 2	0.57	0.23	0.71	0.15	0.174
	Session 3	0.65	0.13	0.66	0.11	0.916

In the control group, both ventilation and TOI values increased significantly from session 1 to session 2 ($p < 0.01$), while the MCI group showed no significant change ($p> 0.05$). Notably, both groups exhibited significant increases in PETCO_2_ between sessions 1 and 2 ($p < 0.005$). This suggests that although both groups experienced elevated PETCO_2_, only the controls responded with compensatory increases in ventilation and cerebral oxygenation, potentially indicating an intact chemoreflex mechanism in the control group that is attenuated in MCI [23]. Post hoc analysis confirmed that controls showed a significant ventilatory increase from Session 1 to Session 2 ($p = 0.006$), while MCI did not ($p = 0.634$). This differential ventilatory response, despite comparable PETCO_2_ elevations in both groups, is consistent with attenuated chemoreflex sensitivity in MCI.

**Fig. 2:**
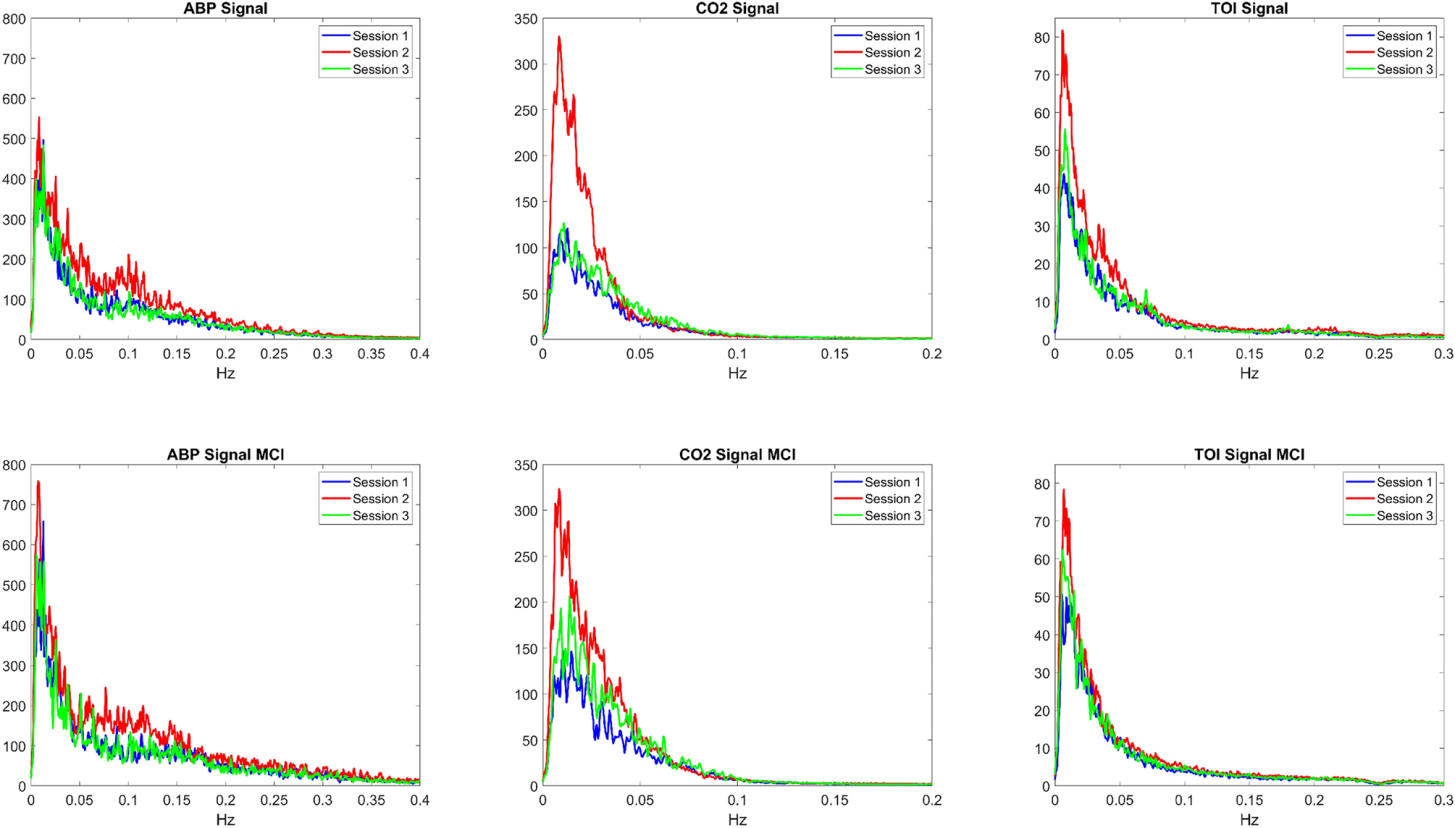
Average spectra of ABP (left), CO$_2$ (middle), and TOI (right) for controls (top) and MCI (bottom) for all 3 sessions

The power spectral density (PSD) plots of the input signals (ABP and PETCO_2_) and the output signal (TOI) for both control and MCI groups are shown in Fig. 2. Across all signals, dominant power resides in the very low-frequency range ($< 0.1$ Hz), consistent with the physiological timescales of cerebrovascular regulation [7, 26]. For ABP, the MCI group consistently exhibits higher spectral power than the controls across all sessions in the very low-frequency range, which may suggest heightened baseline variability or reduced autonomic buffering. Both groups show a subtle increase in ABP spectral power around 0.15 Hz during session 2, potentially reflecting respiratory or baroreflex engagement in response to the gas perturbation [15].

For PETCO_2_, both groups demonstrate a clear increase in spectral power during session 2, as expected from the imposed PRBS gas modulation. However, the control group shows relatively lower spectral power above 0.05 Hz compared to the MCI group, suggesting a more tightly regulated ventilatory response to the stimulus. In session 3, the PETCO_2_ spectrum in the MCI group does not return to its baseline shape and remains elevated across the frequency range, which may reflect impaired CO_2_ clearance or irregular recovery of the chemoreflex response [28].

TOI spectral profiles were broadly similar between groups. However, the control group shows a slight increase in TOI power during session 2 above 0.05 Hz, whereas the MCI group shows little change across sessions. These subtle spectral shifts suggest that while the global dynamics of cerebral oxygenation remain comparable, the control group exhibits a slightly more responsive oxygenation profile during the PRBS gas challenge.

Together, these findings point to subtle but detectable dysregulation in the MCI group’s physiological response to induced gas perturbations, particularly in ABP and CO_2_ dynamics.

**Fig. 3:**
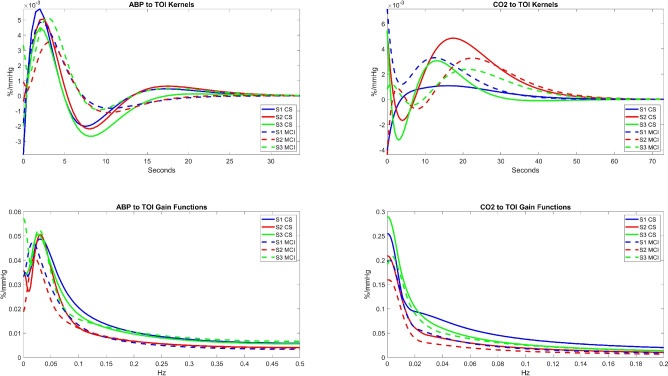
Tissue oxygenation index (TOI) response to PRBS gas perturbation

The average estimated kernels and their corresponding gain functions for ABP and PETCO_2_ inputs are shown in Fig. 3. In the time domain, ABP kernels for both groups exhibit a biphasic profile with a peak followed by an undershoot, characteristic of autoregulatory dynamics. However, the MCI group shows a blunted initial peak and reduced rebound magnitude, suggesting diminished cerebral pressure reactivity. For PETCO_2_, control kernels show a sharp positive peak at approximately 8 s, while MCI kernels display a delayed and more attenuated response.

**Fig. 4:**
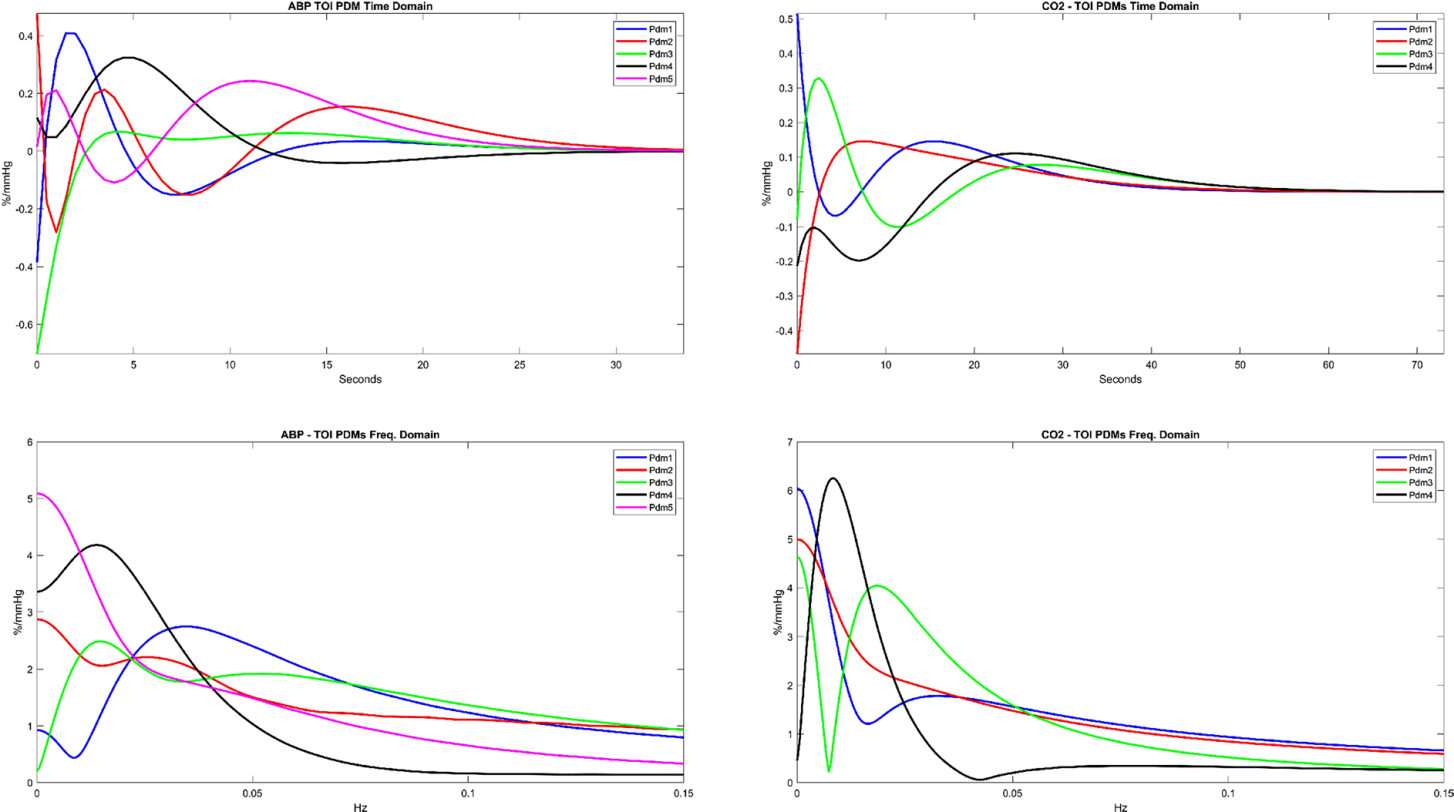
Time-domain waveforms (top) and frequency-domain magnitude spectra (bottom) of the Principal Dynamic Modes extracted from control-group baseline kernels for ABP (left) and CO_2_ (right) inputs. Spectral peaks identify distinct regulatory frequency bands: endothelial/NO-dependent (<0.02 Hz), neurogenic/cholinergic (0.02–0.04 Hz), and myogenic (0.04–0.15 Hz)

Gain analysis further supports this distinction; the spectral profiles of all extracted PDMs are shown in Fig. 4. The first PETCO_2_-derived PDM shows significantly lower Gain values in the MCI group during session 2 ($p = 0.03$), indicating impaired dynamic cerebrovascular response to hypercapnia, Table 2. Significant group-level differences were observed in the fourth ABP-derived PDM, centered at 0.014 Hz. This component was significantly lower in the control group than in MCI during sessions 1 and 3 (*p* < 0.05), indicating higher cerebrovascular resistance at baseline and recovery, Table 3. In session 2, controls showed an elevated Gain in this mode—consistent with transient nitric oxide-mediated vasodilation in response to hypoxic and hypercapnic gas stress—while MCI subjects showed no such modulation. These model-derived features emphasize the potential for computational dynamics to reveal subtle physiological deficits not captured by resting-state averages. RM-ANOVA on CO_2_ PDM 1 Gains confirmed a significant Group $\times$ Session interaction (*p* = 0.016), with no significant group main effect ($p = 0.105$), indicating that the groups diverge in their dynamic response to perturbation rather than differing at baseline. RM-ANOVA confirmed a significant group main effect for ABP PDM 4 ($p = 0.010$), consistent with persistently higher Gains in MCI across sessions.

An additional finding emerged in ABP PDM 5, which showed a significant Session effect ($p = 0.015$) and Group $\times$ Session interaction (*p* = 0.020). In the control group, ABP PDM 5 Gains decreased progressively from Session 1 to Session 3 (S2 vs. S3: $p = 0.014$; S1 vs. S3: *p* = 0.011), while MCI subjects showed no significant session-to-session changes. This pattern may reflect a post-perturbation vascular adaptation that is present in controls but absent in MCI.

**Table 2:** Mean (standard deviation) values of estimated Gains of the PDM outputs for the CO_2_ input and TOI output and the corresponding *p*-values for the two groups

		Controls		MCI		*p*-value
		Mean	SD	Mean	SD	
CO_2_ – PDM 1	Session 1	0.0007	0.045	0.0173	0.018	0.162
	Session 2	0.0238	0.023	0.0073	0.013	**0.016**
	Session 3	0.0131	0.041	0.0097	0.025	0.767
CO_2_ – PDM 2	Session 1	0.0084	0.029	0.0063	0.021	0.811
	Session 2	0.0111	0.015	0.0108	0.013	0.948
	Session 3	0.0041	0.028	0.0053	0.021	0.888
CO_2_ – PDM 3	Session 1	− 0.0010	0.020	0.0031	0.015	0.515
	Session 2	0.0004	0.009	0.0069	0.009	**0.043**
	Session 3	− 0.0089	0.018	0.0049	0.019	**0.036**
CO_2_ – PDM 4	Session 1	0.0025	0.011	− 0.0066	0.019	0.102
	Session 2	0.0081	0.012	0.0121	0.010	0.298
	Session 3	0.0003	0.017	0.0063	0.032	0.509

**Table 3:** Mean (standard deviation) values of estimated Gains of the PDM outputs for the ABP input and TOI output and the corresponding *p*-values for the two groups

		Controls		MCI		*p*-value
		Mean	SD	Mean	SD	
ABP – PDM 1	Session 1	0.0135	0.014	0.0094	0.007	0.2883
	Session 2	0.0113	0.014	0.0050	0.005	0.0893
	Session 3	0.0113	0.010	0.0070	0.007	0.1573
ABP – PDM 2	Session 1	0.0011	0.009	0.0000	0.006	0.6760
	Session 2	0.0045	0.006	0.0023	0.005	0.2568
	Session 3	0.0018	0.009	0.0060	0.011	0.2372
ABP – PDM 3	Session 1	− 0.0009	0.007	− 0.0014	0.004	0.8040
	Session 2	− 0.0022	0.006	− 0.0015	0.006	0.7613
	Session 3	− 0.0022	0.010	− 0.0031	0.011	0.7988
ABP – PDM 4	Session 1	0.0014	0.007	0.0075	0.008	**0.0185**
	Session 2	0.0026	0.005	0.0063	0.006	0.0516
	Session 3	0.0002	0.009	0.0087	0.007	**0.0042**
ABP – PDM 5	Session 1	− 0.0006	0.003	− 0.0021	0.005	0.3246
	Session 2	− 0.0018	0.004	− 0.0038	0.004	0.1540
	Session 3	− 0.0048	0.005	− 0.0013	0.007	0.1128

## Discussion

This study presents the first application of a pseudorandom binary sequence (PRBS) breathing protocol to investigate dynamic cerebrovascular regulation in individuals with mild cognitive impairment (MCI). The broadband spectral content of PRBS-induced perturbations enabled the detection of physiologically meaningful dynamics that remain obscured under spontaneous or steady-state conditions. All participants completed the protocol without difficulty or adverse effects, highlighting its safety and tolerability. Compared to traditional intermittent gas exposure methods, PRBS breathing offers a more compact and controlled stimulus for probing cerebrovascular responses [29]. Furthermore, all subjects were ApoE4-negative to isolate effects of cognitive status from the well-documented cerebrovascular impact of the ApoE4 genotype [18].

Spectral analysis of the raw signals further differentiated the groups. MCI subjects demonstrated higher baseline low-frequency ABP power across sessions, consistent with reduced autonomic buffering. PETCO_2_ spectral power increased during PRBS in both groups, but returned to baseline in controls by session 3 while remaining elevated in MCI—suggesting impaired CO_2_ clearance or persistent chemoreflex activation. These spectral differences support a picture of preserved homeostatic recovery in controls but sustained dysregulation in MCI.

Dynamic modeling allowed us to move beyond group mean differences and probe the time- and frequency-dependent structure of cerebrovascular responses. By modeling TOI as an output of both ABP and PETCO_2_ inputs, we were able to assess autoregulatory, chemoreflex, and cholinergic-linked mechanisms under perturbation. These models revealed distinct regulatory dynamics between groups that were not apparent from resting physiological measures alone.

Kernel and Gain function analysis deepened these findings. ABP kernels in controls exhibited a delayed negative deflection that increased across sessions—consistent with baroreflex-mediated vascular resistance modulation. This feature was diminished or absent in MCI, indicating impaired dynamic autoregulation. PETCO_2_ kernels increased substantially in controls during PRBS exposure and remained elevated in session 3, suggesting a persistent or adaptive cerebrovascular response. MCI subjects, by contrast, exhibited only transient increases in PETCO_2_ kernel magnitude.

These findings are consistent with prior cerebrovascular modeling studies. Our NMSE values (0.57–0.71) are comparable to those reported in Marmarelis et al. [22, 24] for similar NIRS-based models. The ABP kernel time scales (peak at $\sim$3 s, undershoot at $\sim$8 s) align with autoregulatory impulse response functions characterized in Claassen et al. [7]. The CO_2_ PDM frequency peaks ($\sim$ 0.019 Hz and $\sim$ 0.033 Hz) correspond to bands identified in Kvandal et al. [16]. Critically, the present study uses active PRBS perturbation rather than spontaneous fluctuations, which enhances signal-to-noise in kernel estimation and may explain the stronger group differentiation observed here.

Principal Dynamic Mode (PDM) analysis provided a global, frequency-resolved perspective, with the spectral profiles of the extracted PDMs shown in Fig. 4. The fourth ABP-derived PDM, centered at approximately 0.014 Hz, was significantly elevated in controls during session 2 only—possibly reflecting transient endothelial-dependent vasodilation under gas stress. Its absence in MCI suggests reduced capacity to modulate vascular resistance dynamically. The time-domain profile of this mode, which is predominantly positive, aligns with known patterns of cerebral vascular admittance. ABP PDM 5 exhibited a significant Group $\times$ Session interaction ($p = 0.020$), with controls showing progressive Gain decreases from Session 2 to 3 ($p = 0.014$) that were absent in MCI. This finding suggests an additional dimension of post-perturbation vascular adaptation that is compromised in MCI.

Two CO_2_-derived PDMs further differentiated the groups. The first CO_2_ PDM, a low-pass integrative mode with a mild resonance near 0.033 Hz, showed a marked Gain increase in session 2 for the control group, consistent with chemoreflex activation in response to the gas challenge [27]. In contrast, the MCI group exhibited a slight decrease in Gain, resulting in a significant group difference during the stimulation period. This divergence suggests that while both groups experienced comparable elevations in PETCO_2_, only the controls engaged the corresponding regulatory mechanisms effectively.

The third CO_2_ PDM, with a strong resonance near 0.019 Hz,operating in a frequency range previously attributed to neurogenic or cholinergic vascular regulation [16, 23], exhibited sustained Gain elevation in controls across sessions 2 and 3 but remained flat in MCI. This prolonged response may reflect a neuroplastic adaptation in healthy individuals, potentially mediated by cholinergic pathways involving the basal forebrain, a region known to degenerate early in dementia [3, 6, 9]. Given the basal forebrain’s dual role in cortical projection and cerebrovascular regulation, these findings may indicate early disruption of dynamic cholinergic-vessel coupling in MCI.

Taken together, these results support six major conclusions:

1. Gas-induced vasodilation, reflected in increased ABP PDM 4 Gain, occurred in controls only during session 2, consistent with transient endothelial-dependent reduction in cerebrovascular resistance.
2. Impaired autoregulation was evident in MCI, marked by absent ABP kernel adaptation and flat PDM 4 Gains across sessions.
3. Chemoreflex activation was captured in CO_2_ PDM 1, which increased significantly during PRBS exposure in the control group but decreased slightly in MCI, indicating impaired downstream signal integration from primary chemoreflex activation.
4. A dynamic mode in the frequency range associated with cholinergic regulation (CO_2_ PDM 3) was present and sustained in controls but absent in MCI, possibly implicating basal forebrain dysfunction in impaired individuals.
5. Raw PETCO_2_ spectra showed incomplete recovery in MCI during session 3, suggesting reduced gas clearance or prolonged chemoreflex engagement.
6. Neuroplastic responses, evidenced by persistent changes in ventilation, TOI, and PETCO_2_ dynamics in controls, were not observed in MCI–indicating a loss of adaptive flexibility in cerebrovascular control.

These findings collectively support the hypothesis that MCI is associated with dysfunction across multiple layers of cerebrovascular regulation—including blunted baroreflex adaptation, reduced chemoreflex-linked ventilation, and impaired cholinergic support of vascular tone. Notably, these deficits were only revealed through the use of broadband perturbation and dynamic modeling, underscoring the limitations of resting-state assessments in identifying early dysfunction.

By combining PRBS breathing with Laguerre-based system identification and PDM decomposition, this study offers a sensitive framework for uncovering latent physiological impairments in early cognitive decline. This approach enables mechanistic interpretation of cerebrovascular dynamics and may support future efforts in biomarker development, stratification of at-risk individuals, and tracking of therapeutic interventions.

Several alternative explanations for the persistent PETCO_2_ spectral elevation in MCI during Session 3 warrant consideration. These include: slower autonomic recovery, supported by the higher baseline low-frequency ABP power and the ABP PDM 5 interaction findings; differences in metabolic CO_2_ production; and the possibility that the 10-min recovery period was insufficient for complete chemoreflex washout in MCI subjects [2]. Notably, mean PETCO_2_ values returned to comparable levels by Session 3 (Controls: 35.24, MCI: 33.65, $p = 0.394$), suggesting the spectral elevation reflects altered temporal dynamics rather than sustained absolute differences.

### Limitations

This study has several limitations that should be considered when interpreting the findings. First, the sample size ($n = 17$; 9 controls, 8 MCI) is modest, limiting statistical power and generalizability; the results should be considered hypothesis-generating. Second, this is a single-site study, and findings may not generalize to other clinical populations. Third, the ApoE4-negative genotype requirement, while scientifically motivated, excludes a significant proportion of MCI/AD populations. Fourth, NIRS-derived TOI captures cortical oxygenation dynamics but does not directly measure cerebral blood flow velocity, which may provide complementary information. Fifth, the sequential, non-counterbalanced session design means that carry-over effects from the PRBS session could influence recovery dynamics observed in Session 3. Sixth, first-order Volterra modeling was used; nonlinear interactions between ABP and CO_2_ inputs were not captured and may contribute additional explanatory variance. Finally, the 10-min recovery period may be insufficient for complete cerebrovascular recovery, particularly in MCI subjects with impaired regulatory capacity.

## Data Availability

The datasets used and analyzed during the current study are available from the corresponding author on reasonable request.

## Declarations

### Clinical Trial Registration

Clinical trial number: not applicable.
